# Total and specific serum IgE decreases with age in patients with allergic rhinitis, asthma and insect allergy but not in patients with atopic dermatitis

**DOI:** 10.1186/1742-4933-2-9

**Published:** 2005-05-31

**Authors:** Anja Mediaty, Karsten Neuber

**Affiliations:** 1 Department of Dermatology, University Hospital Eppendorf, Martinistr. 52, 20246 Hamburg, Germany

## Abstract

Concerning allergic diseases, the incidence of allergic symptoms, as well as their severity, seems to decrease with age. The decline of onset of allergic symptoms observed in ageing might result from a decrease of serum total and specific IgE. Atopic disorders are complex diseases that involve interactions among several physiological systems, e.g. skin, lung, mucosae, and the immune system. It was the aim of this study to compare the effects of age on total and specific IgE in patients with atopic dermatitis (AD), allergic rhinitis or asthma, and insect allergy, respectively.

The study population consisted of 559 individuals (male: 229 and female: 330). Total and allergen specific IgE was measured in every individual. From the whole study population, 113 patients suffered from atopic dermatitis (AD), 132 had allergic rhinitis or asthma, and 314 were tested because of insect allergy. Total and specific serum IgE was significantly decreased as a function of age in patients with allergic rhinitis and asthma and with insect allergy. In contrast, no significant decrease of total and specific serum IgE in old individuals with AD was observed. Additionally, in the group of patients with a total IgE < 300 kU/l a reduction of total serum IgE was significantly correlated with age. In contrast, patients with IgE levels > 300 kU/l showed no correlation with age.

Immunosenescence does not affect increased IgE levels in atopic patients with AD and/or high serum IgE levels indicating that in these subgroups of patients the atopic propensity remains into advanced age. One may hypothesize that either onset of allergic sensitization during life or the kind of atopic disease influences the correlation between age and IgE synthesis.

## Introduction

Concerning allergic diseases, the incidence of onset of allergic symptoms, as well as their severity seems to decrease with age [1]. Atopy is a relatively common, adverse humoral immune system response to common environmental agents (allergens) involving the production of allergen-specific IgE. Epidemiological investigations of allergen sensitivity in a community-based population [2-5] and an industrial setting [6,7] show that the propensity for and the relative incidence of atopic disorders tend to change with age. Serum total IgE values decline with age in the general population, and there are significantly fewer cases of atopy among elderly subjects (60 years and older) compared with younger subjects. However, atopic disorders are complex diseases that involve interactions among several physiological systems, e.g. skin, lung, mucosae, and the immune system.

The immune system undergoes characteristic changes with ageing. Most tests of T cell function are depressed in elderly individuals and the deterioration of the immune systems is believed to contribute to morbidity and mortality in man [8].

Age-associated alterations in the proportions of T cell subsets have been well documented in humans. There are clearly more CD4+ CD45RO+ "memory-phenotype" cells and less CD45RA+ "naïve-phenotype" cells in peripheral blood mononuclear cells (PBMC) from elderly individuals. The accumulation of CD45RO+ memory cells results in a reduced ability to respond to new antigens, and a retained ability to respond to recall antigens, as long as the memory cells remained present and functional [9-11].

Data on age-associated alterations in cytokine secretion in human are inconsistent. Th1 cells are characterised by their ability to secrete IFNγ, IL-2, TNF-α, IL-12 and IL-15, whereas Th2 cells are characterised by IL-4, IL-5, IL-6, IL-10 and IL-13 secretion. Infant humans exhibit impaired cellular but strong humoral immunity, and are in a type 2-dominant state. Soon thereafter, a type 1-state becomes dominant and persists in healthy humans until mid-to-later life, at which time a dominant type 2 cytokine profile may again emerge [12]. Paradoxically, despite declining T cell function, ageing does nor appear always to be associated with decreased immune reaction. For instance, there are increased levels of autoantibodies in the aged [13], resulting in autoimmune diseases (e.g. bullous pemphigoid).

Current thought posits that age-associated changes in B-cell function are primarily due to changes in the T-cell compartment and a resultant dysregulation on B-cell function [14]. Altered function is primarily associated with exposure to "new" antigenic challenges, as for example the less efficient therapeutic value of inoculation regimes among the elderly population. Although both T cells and B cells are involved with the onset of atopy, the effector mechanisms are principally humorally driven and focused on the production and regulation of IgE. To what extent this regulation is T cell dependent or T cell independent is not well stablished. Recently, it has been demonstrated that in healthy non-atopic individuals no differences in either serum total IgE and soluble CD23 or Th2 related cytokines (IL-4, IL-10 and IL-13) between young and old subjects were observed [15].

However, there are not many studies investigating the relationship between age and IgE in different atopic diseases. In this study, we evaluated the effects of age on total and specific IgE in patients with atopic dermatitis (AD), allergic rhinitis or asthma, and insect allergy.

## Methods

### Study population

The population of this retrospective study consisted of 559 individuals (male: 229 and female: 330) randomly selected from a data bank of patients diagnosed for one of the relevant diseases. The diagnoses were made on the basis of the case history and the clinical examination. Total and/or allergen specific IgE was measured in every individual. From the whole study population, 113 patients suffered from AD, 132 had allergic rhinitis or asthma, and 314 were tested because of insect allergy.

### Determination of serum IgE

Total and allergen specific serum IgE was measured by using the UniCAP System (Pharmacia, Freiburg, Germany) according to the instructions of the manufacturer. Directly after collection of venous blood, the blood was centrifuged and serum was stored at -20°C until testing. Total serum IgE levels < 300 kU/l are in the normal or borderline range, amounts ≥ 300 kU/l were classified as pathological [17]. Serum IgE specific was determined at least for one of the following antigens: Dermatophagoides pteronyssinus and farinae (Der p 1 and Der f 1), Betula verrucosa (Bet v 1), Phleum pratense (Phl p 1), Felis domesticus (Fel d 1), and hymenoptera venoms. In patients with allergic asthma or rhinitis and in patients with AD Der p 1 and Der f 1, Bet v 1, Phl p 1, and Fel d 1 were measured. In patients with insect allergy specific IgE to hymenoptera venoms was tested. For patients polysensitized against several allergens, the highest value of specific IgE was selected and only this value was used for statistical analysis.

### Statistical analysis

Statistical analysis for paired comparisons of total and specific IgE between different patient groups was conducted by Student's *t *test. In order to assess the relationship between IgE and age, we calculated Kendall's tau-b and the corresponding *p*-values to test the hypothesis that both variables are not associated [18]. Additionally, we used simple linear regression to evaluate age-associated changes in serum IgE. Statistical analysis was performed by using the software program SPSS 9.0.

## Results

### Patients characteristics

**Figure 1:**
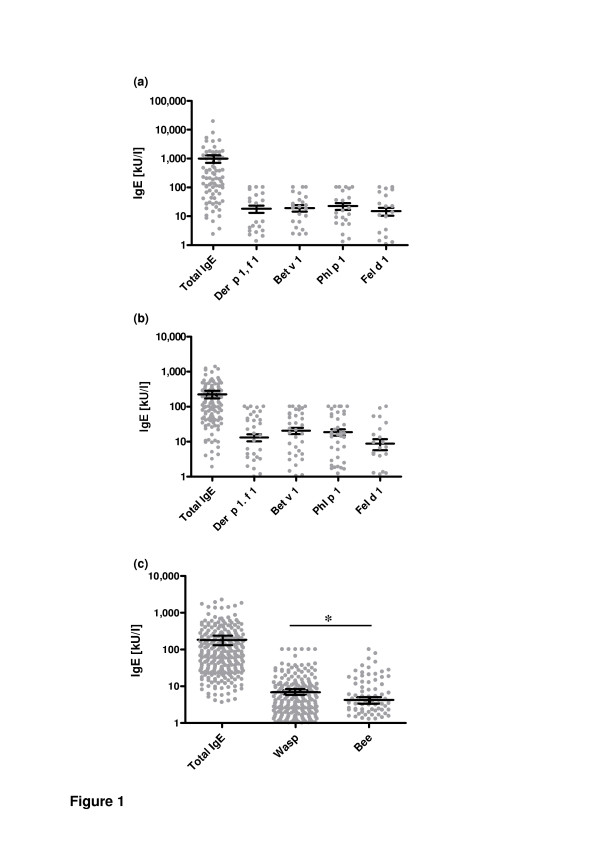
**Total and specific IgE levels**. Means ± SEM of total and specific IgE [kU/l] for Der p 1/f 1, Bet v 1, Phl p 1, and Fel d 1 in patients with (a) AD and with (b) allergic asthma or rhinitis as well as for (c) wasp and bee allergen in patients with insect allergy are shown. Total and specific IgE is sicnificantly increased in patients with AD compared with patients suffering from allergic asthma or rhinitis and insect allergy.

The study population consisted of 559 patients suffering from either AD (n = 113), allergic asthma or rhinitis (n = 132) or insect allergy (n = 314) tested for total and specific serum IgE (Table 1). Mean age of patients with AD was 23.5 years, whereas mean age of the patients with allergic rhinitis, asthma or insect allergy was 36.7 years and 44.2 years, respectively (Table 1). In the group of patients > 65 year old 6 (5.3%) had AD, 24 (18.2%) sufferd from rhinitis or asthma, and 105 (33.4%) from insect allergy. Twentytwo patients with insect allergy were younger than 10 years. In the group of patients with insect allergy older than 65 years the onset of symptoms was always over 60 years but not in the group of patients with AD and allergic rhinitis or asthma where symptoms onset always started earlier. Independent of age, the highest amounts of total and specific IgE (Figure 1) were found in patients with AD compared with the other patient groups (p < 0.01; p < 0.02).

**Table 1:** Correlation between age and IgE. Characteristics of patients and Kendall's tau-b for relationship between IgE and age.

	Mean Age ± SD (range)	Age (yr)		Number of patients (%)		Total IgE [kU/l] vs Age			Specific IgE [kU/l] vs Age		
		>65	>65	male	female	τ_b_	(n)	*p*	τ_b_	(n)	*p*
All patients	36.9 ± 22.6 (0.5 – 93)	424	135	229 (40.9)	330 (59.0)	-0.262	(553)	< 0.0001	-0.176	(485)	< 0.0001
Atopic dermatitis	23.6 ± 20.1 (0.5 – 93)	107	6	53 (46.9)	60 (53.1)	-0.038	(112)	= 0.561	0.034	(68)	= 0.696
Allergic Rhinitis/Asthma	36.7 ± 19.6 (3.0 – 79)	108	24	50 (37.9)	82 (62.1)	-0.219	(132)	< -0.0001	-0.320	(107)	< 0.0001
Insect Allergy	44.2 ± 21.7 (2.0 – 82)	209	105	126 (40.1)	188 (59.9)	-0.259	(309)	< 0.0001	-0.084	(310)	= 0.029

### Ageing and total serum IgE

The total IgE had a negative correlation (p < 0.0001) with age in all patients as well as in the subgroups of patients with allergic rhinitis and asthma, and insect allergy. In contrast, no significant decrease of total serum IgE as a function of age was observed in patients with AD (Table 1).

**Figure 2:**
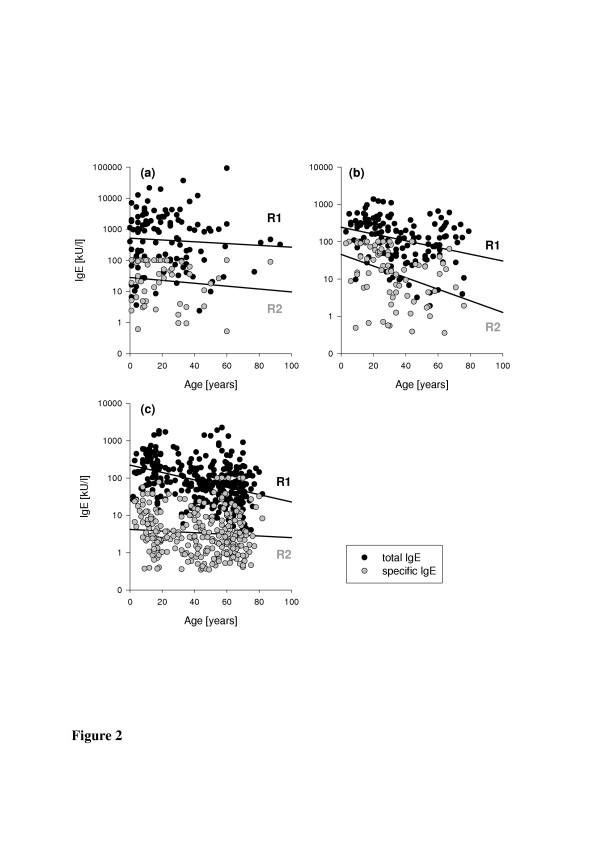
**Correlation between age and IgE**. Multiple scatter plots of donor age versus total (●) and specific (○) serum IgE. (a) patients with AD, (b) patients with allergic asthma or rhinitis; (c) patients with insect allergy. R1, line for total IgE and R2 for specific IgE derived from simple linear regression.

Figure 2 shows the values of total serum IgE (kU/l) against donors age for patients with AD, allergic asthma or rhinitis, and for insect allergy. For patients with allergic asthma and rhinitis (*F*_(1,130) _= 10.850, *p *= 0.001) as well as for patients with insect allergy (*F*_(1,307) _= 16.236, *p *<0.0001), the slopes of the lines derived from simple linear regression do vary significantly from zero, but do not for patients with AD (*F*_(1,110) _= 1,734, *p *= 0.191).

Additionally, in the group of patients with a total IgE < 300 kU/l a reduction of total serum IgE was significantly correlated with age (*p *<0.0001). In contrast, patients with IgE levels > 300 kU/l showed no correlation with age (Table 2). From the patients with IgE levels > 300 kU/l, 16 (11.9%) were over 65 years old. Linear regression analysis revealed *F*_(1,400) _= 20.955 (*p *<0.0001) for total IgE levels < 300 kU/l but were not significant for IgE levels > 300 kU/l (*F*_(1,149) _= 1,138, *p *= 0.288)

**Table 2:** Correlation between age and IgE. Kendall's tau-b for relationship between IgE (< 300 kU/l or > 300 kU/l) and age.

Total IgE	Mean Age ± SD (range)	Age (yr)		Number of patients (%)		Total IgE [kU/l] vs Age		
		< 65	> 65	male	female	τ_b_	(n)	p
< 300 kU/l	42.9 ± 21.4 (1.0 – 82)	274	118	148 (36.8)	254 (63.2)	-0.151	(402)	< 0.0001
> 300 kU/l	26.0 ± 20.1 (0.5 – 93)	151	16	80 (53.0)	71 (47.0)	-0.055	(151)	= 0.320

### Ageing and allergen specific IgE

Figure 1 shows specific IgE values for each tested allergen in the different disease groups. No significant differences of mean IgE values between the different allergens could be detected except in patients with insect allergy. In patients with insect allergy only wasp and bee allergen were tested and the mean specific IgE for bee was significantly (p = 0.02) lower compared with wasp specific IgE. The majority of these patients (91.7%) had immunotherapy because of wasp allergy, whereas 8.3% had immunotherapy because of clinical relevant sensitization (Table 3). In patients with atopic dermatitis or with rhinitis/allergic asthma relevant sensitization (0.35 kU/l) against the different allergens was distributed homogenously. Only sensitization to Fel d 1 was less frequent in patients with allergic rhinitis or asthma (Table 3).

**Table 3:** Frequencies of allergens in the disease groups. Number of subjects in each disease group who were positive at ≤ 0.35 kU/l to the tested allergens.

Disease	Atopic Dermatitis	Allergic Rhinitis and Asthma	Insect Allergy
Allergen	(n = 113)	(n = 132)	(n = 314)
Der p 1 and f 1	26 (23.0%)	39 (29.5%)	--
Bet v 1	27 (23.9%)	43 (32.6%)	--
Phl p 1	24 (21.2%)	40 (30.3%)	--
Fel d 1	22 (19.5%)	23 (17.4%)	--
Wasp	--	--	288 (91.7%)
Bee	--	--	26 (8.3%)

Allergen specific IgE was significantly decreased in the elderly suffering from allergic rhinitis, allergic asthma (*p *< 0.0001) and insect allergy (*p *= 0.029), respectively. On the other hand, no correlation between specific IgE and age was observed in patients with AD (Table 1).

For patients with allergic asthma and rhinitis (*F*_(1,130) _= 26.437, *p *= 0.0001) the slopes of the lines derived from simple linear regression do vary significantly from zero (Figure 2). For patients with insect allergy (*F*_(1,308) _= 0.110, *p *= 0.740) and for patients with AD (*F*_(1,68) _= 0,283, *p *= 0.596), linear regression did not reveal significant variation from zero for allergen specific IgE (Figure 2).

## Discussion

From a clinical perspective, supported by epidemiological investigations, there would appear to be a decline with age in both the incidence and severity of atopic diseases, particularly among the elderly population who are 60 years and older [2-7]. Atopic incidence declines, symptoms severity declines, and there would appear to be a general humoral alteration of the propensity for atopy reflected by age-associated declines in serum total IgE values. However, the results of this study demonstrate that the association between age and serum IgE is dependent on the type of atopic or allergic disease and is also dependent on the amount of total serum IgE.

The results of our study are supported by data that have been published recently by Jackola et al. [16]. In patients with allergic asthma they observed a decline with age in serum total IgE values. Moreover, they showed that among those asthmatic individuals sensitized to ragweed antigen, there was no age-associated change in IgE levels specific to the major ragweed allergen. This robustness of the underlying atopic mechanism, represented by specific IgE was also observed in our patients with AD but not in the patients with allergic asthma or rhinitis and in patients with insect allergy. Concerning insect allergy, this difference might be partly explained by the later onset of sensitization against hymenoptera venoms during life and thus, other immunological mechanisms regulating the production of specific IgE in patients with insect allergy, which has been defined as hypersensitivity allergic nonatopic IgE-mediated disease [17]. This assumption is supported by the fact that in the group of patients with insect allergy older than 65 years the onset of symptoms was always over 60 years but not in the group of patients with AD and allergic rhinitis or asthma.

The differences according age-association of allergen-specific IgE in patients with asthma between the two studies might be the consequence of patient's selection. The subgroup of patients with asthma in our study probably was more heterogenous compared with the population investigated by Jackola and co-workers. Moreover, it is not clear whether asthma patients with additional manifestations of other atopic diseases (e.g. AD or rhinitis) were excluded in their study or not.

As expected, the numbers of patients with AD older than 60 years were rather small. However, a higher proportion of AD patients were over 40 years old indicating that in this group of adults IgE production is unaffected by age.

The results of our study support the assumption that age-association of total and specific serum IgE is dependent on the type of atopic disease the patients are suffering from and the age at disease onset.

## Conclusion

In summary, this study shows that total and allergen specific IgE production is reduced in the elderly with the exception of old patients with either high serum IgE or AD, indicating that atopic mechanisms underlying AD or other atopic diseases with high serum IgE are particularly robust, and the atopic propensity among these patients remains into advanced age. However, further studies are required to clarify the immunological mechanisms which are responsible for IgE synthesis during immunosenscence.

## Competing interests

The author(s) declare that they have no competing interests.

## Authors' contributions

AM participated in the design of the study, performed the statistical analysis and drafted the manuscript. KN conceived of the study, and participated in its design and coordination and helped to draft the manuscript. All authors read and approved the final manuscript.

## References

[B1] Hanneuse Y, Delespesse G, Hudson D, de Halleux F, Jacques JM (1978). Influence of ageing on IgE-mediated reactions in allergic patients. Clin Allergy.

[B2] Barbee RA, Brown WG, Kaltenborn W, Halonen M (1981). Allergen skin-test reactivity in a community population sample: correlation with age, histamine skin reactions and total serum immunoglobulin E. J Allergy Clin Immunol.

[B3] Barbee RA, Halonen M, Lebowitz M, Burrows B (1981). Distribution of IgE in a community population sample: correlations with age, sex, and allergen skin test reactivity. J Allergy Clin Immunol.

[B4] Barbee RA, Halonen M, Kaltenborn W, Lebowitz M, Burrows B (1987). A longitudinal study of serum IgE in a community cohort: correlations with age, sex, smoking, and atopic status. J Allergy Clin Immunol.

[B5] Barbee RA, Kaltenborn W, Lebowitz MD, Burrows B (1987). Longitudinal changes in allergen skin test reactivity in a community population sample. J Allergy Clin Immunol.

[B6] Freidhoff LR, Meyers DA, Bias WB, Chase GA, Hussain R, Marsh DG (1981). A genetic-epidemiologic study of human immune responsiveness to allergens in an industrial population: I. Epidemiology of reported allergy and skin-test positivity. Am J Med Genet.

[B7] Freidhoff LR, Meyers DA, Marsh DG (1984). A genetic-epidemiologic study of human immune responsiveness to allergens in an industrial population. II. The associations among skin sensitivity, total serum IgE, age, sex, and the reporting of allergies in a stratified random sample. J Allergy Clin Immunol.

[B8] Pawelec G, Barnett Y, Forsey R, Frasca D, Globerson A, McLeod J, Caruso C, Franceschi C, Fulop T, Gupta S, Mariani E, Mocchegiani E, Solana R (2002). T cells and aging, January 2002 update. Front Biosci.

[B9] Hannet I, Erkeller-Yuksel F, Lydyard P, Deneys V, Bruyere MD (1992). Developmental and maturational changes in human blood lymphocyte subpopulations. Immunol Today.

[B10] Gabriel H, Schmitt B, Kindermann W (1993). Age-related increase of CD45RO+ lymphocytes in physically active adults. Eur J Immunol.

[B11] Flurkey K, Stadecker M, Miller RA (1992). Memory lymphocyte-T hyporesponsiveness to non-cognate stimuli - a key factor in age-related immunodeficiency. Eur J Immunol.

[B12] Shearer GM (1997). Th1/Th2 changes in aging. Mech Ageing Dev.

[B13] Klinman NR, Kline GH (1997). The B-cell biology of aging. Immunol Rev.

[B14] LeMaoult J, Szabo P, Weksler ME (1997). Effect of age on humoral immunity, selection of the B-cell repertoire and B-cell development. Immunol Rev.

[B15] Di Lorenzo G, Pacor ML, Esposito Pellitteri M, Listi F, Colombo A, Candore G, Mansueto P, Lo Bianco C, Ditta V, Battista Rini G, Caruso C (2003). A study of age-related IgE pathophysiological changes. Mech Ageing Dev.

[B16] Kendall M, Gibbons JD (1990). Rank Correlation Methods.

[B17] Jackola DR, Pierson-Mullany LK, Daniels LR, Corazalla E, Rosenberg A, Blumenthal MN (2003). Robustness into advanced age of atopy-specific mechanisms in atopy-prone families. J Gerontol A Biol Sci Med Sci.

[B18] Johansson SG, Hourihane JO, Bousquet J, Bruijnzeel-Koomen C, Dreborg S, Haahtela T, Kowalski ML, Mygind N, Ring J, van Cauwenberge P, van Hage-Hamsten M, Wuthrich B (2001). A revised nomenclature for allergy. An EAACI position statement from the EAACI nomenclature task force. Allergy.

